# New concepts on abnormal UV reactions in systemic lupus erythematosus and a screening tool for assessment of photosensitivity

**DOI:** 10.1111/srt.13247

**Published:** 2023-03-06

**Authors:** Danielle Corbin, Lea Christian, Christine M. Rapp, Langni Liu, Craig A. Rohan, Jeffrey B. Travers

**Affiliations:** ^1^ Department of Pharmacology & Toxicology Boonshoft School of Medicine at Wright State University Dayton Ohio USA; ^2^ Department of Dermatology Boonshoft School of Medicine at Wright State University Dayton Ohio USA; ^3^ Department of Medicine (Dermatology) Dayton Veterans Administration Medical Center Dayton Ohio USA

Dear Editors,

Systemic lupus erythematosus (SLE) is a multisystem autoimmune recurrent inflammatory condition that most often manifests as a mixture of mucocutaneous, musculoskeletal, and hematologic involvements. SLE is subdivided into acute, subacute, and chronic forms.^1^, ^2^ As many as 90% of SLE patients develop mucocutaneous symptoms at some point during the course of their disease, and up to 25% have cutaneous involvement at the time of diagnosis.^1^, ^3^ The presentation of mucocutaneous symptoms range in severity and frequency and are often triggered and intensified by ultraviolet radiation (UVR).^3^ The resulting cutaneous lesions can occur with or without SLE.^3^

SLE can run many courses clinically and as such has a classification that separates the 47 forms that have cutaneous involvement from the overall systemic involvement of the disease. SLE Diagnostic Criteria, as described by the American College of Rheumatology (ACR) and European League Against Rheumatism in 2019, pose the new criteria that appear to have an increased specificity and sensitivity compared to previous diagnostic criteria models.^4^

SLE affects 62.2–84.8 per 100 000 in the United States with an estimated prevalence of 4.6–5.6 per 100 000 per year.^2^ SLE is 3–4× more prevalent in Black women, 1.5–2× more likely in Asian and Hispanic women than in White women, with evidence that there are disparities due socioeconomic factors that play a role in the diagnosis and treatment of SLE.^5^, ^6^ Black women tend to develop SLE earlier in life and have a higher mortality rate.^6^ The risk for skin damage is higher in Black people with lupus than in Whites and Hispanics.^6^, ^7^ The incidence of cutaneous lupus erythematosus (CLE) is 4 cases per 100 000, and the prevalence is 73 cases per 100 000 persons.^8^ SLE is not a reportable diagnosis, and thus, it is likely that number of people with this condition is greater than estimated.

Broadly, photosensitivity (PSN) is an abnormal reaction to sunlight/UVR exposure that leads to a rash or lesion formation as determined by a practitioner. Photosensitivity is one of the 11 classification criteria for SLE, and the ACR defines it as “a skin rash as a result of unusual reaction to sunlight.” Between 23% and 83% of patients with SLE are reported to be sensitive to UVR.^3^ True photosensitive reactions to UVR can occur up to 3 weeks after exposure and may present with fatigue and arthralgias.^2^, ^3^

Following UV exposure, skin reactions are often delayed (more than a week after testing). It is critical that practitioners ask patients about recent prolonged exposures and symptoms such as fatigue, especially toward the end of the summer season. Patients often mistake these rashes for sunburn, acne, or rosacea, which further delays treatment.^1^, ^3^, ^8^, ^9^ Ensuring patient understanding of the risks related to unprotected sun exposure can help reduce the skin lesion formation, exacerbation, and further systemic involvement. Based on these findings, we support expanding the definition of photosensitivity to include a skin rash or lesion that appears in a delayed mechanism, days to weeks after excessive sun exposure and persisting further for days to weeks.

Regarding the mechanisms for the photosensitivity associated with lupus erythematosus, several recent reviews have been published.^10^, ^11^ In this review, we will summarize the relationship of photosensitivity, with a particular emphasis on CLE lesion formation, and provide novel preclinical murine data suggesting that subcellular microvesicle particles (MVP) could play a role in the photosensitivity response. Finally, we propose a simple tool to categorize photosensitivity in patients.

Adaptive skin pigmentation is a normal protective measure and the skin's first line of defense against UVR. Typically, UV light induces the activation of repair mechanisms while delaying keratinocyte cell growth.^12^, ^13^ Keratinocytes and melanocytes coordinate the increased production of melanin that is used to protect neighboring cells from UV penetration, which damages DNA.^14^ Irreparable cell damage from UVR leads to apoptosis via the Fas and FasL pathways in keratinocytes (a.k.a. “sunburn cells”) in the epidermis.^14^, ^15^ Over the next few days, these cells are shed via the stratum corneum or phagocytosed by skin resident macrophages, including Langerhans cells (LCs).

There is evidence from studies using animal models that both increased numbers and incomplete clearing of apoptotic cells predisposes to SLE and autoimmunity, though the complete pathogenesis in human skin is still being elucidated.^16^ The increased keratinocyte apoptosis in SLE patients in response to UVR suggests a defective clearance of apoptotic cell debris increases auto‐reactivity of lymphocytes. One potential mechanism involves the increased type I interferon genes that decrease numbers and activity of regulatory T‐cells.^17^

Ultraviolet rays (UVA and UVB) penetrate the epidermis and dermis and contribute to cutaneous lesion formation and apoptotic pathways in the skin though via potentially different mechanisms.^3^, ^15^, ^16^ UVB induces the direct damage of DNA through the formation of pyrimidine dimers and can activate the p53‐dependent cell death pathway. UVB radiation triggers CD95‐dependent cell death via increased receptor aggregation independently of CD95L.^3^ Following UVB exposure, increased cytokines and immunomodulatory factors have been measured in the skin, including interleukin‐1 (IL‐1), tumor necrosis factor‐α (TNF‐α), intracellular adhesion molecule‐1, and histocompatibility class II molecules (e.g., HLA‐DR3).^18^ In contrast, UVA has been associated with causing mitochondrial damage and increasing reactive oxygen species (ROS) which ultimately induce cell death via the mitochondrial pathway.^3^, ^15^

Recent studies have further established the connection between photosensitivity and SLE, and mucocutaneous involvement has a 75%–80% likelihood of presenting in patients over the course of their disease progression.^3^ The incidence of photosensitivity may be assumed to be even higher amongst patients with lupus, but many of the systemic therapies used empirically or early in the course of patients with lupus, including hydroxychloroquine and azathioprine, likely decrease the incidence of mucocutaneous features. CLE is a subset of SLE that characterizes the various skin involvements into the acute, subacute, and chronic types. CLE does not have a uniform definition, and diagnosis is a result of the identification of certain clinical findings, location and duration of lesions, serum levels, pathological findings, and immunofluorescence results.^1^, ^19^

Photoprovocation has been used to induce lesion formation in patients while studying the cutaneous manifestations of SLE. There is no standard procedure for reproducing or reporting provocative phototesting of CLE lesions, and a variety of techniques have been reported.^4^, ^20^, ^21^, ^22^ Researchers may choose to enroll study subjects who meet the ACR CLE or SLE diagnostic criteria for evaluation and participation. Phototesting results do not regularly coordinate with a positive history of photosensitivity, and those with a negative history of photosensitivity report positive phototesting results in more than 50% cases.^3^, ^22^ Photosensitivity can also be induced by drug reactions such as fluoroquinolone antibiotics.^23^ The lack of standardized protocol exemplifies the challenges in characterizing and quantifying photosensitivity in patients with CLE.

UVA radiation can be divided into UV‐A1 (340–400 nm) and UV‐A2 (320–340 nm) lights. UV‐A1 has similar properties to visible light and has overall lesser pathologic effects in the skin due to its radiation not being absorbed by DNA. UV‐A1 radiation in mice showed a reduction of spleen enlargement, anti‐dsDNA antibody levels, and increased lymphocyte mitogen responsiveness. In humans, UV‐A1 decreased disease activity according to patient systemic lupus activity scores that assessed levels of fatigue, depression, cognitive dysfunction, joint pain, and mouth ulcers. Typical photosensitive lesion formation was also delayed.^24^

UVR is implicated in the production and recruitment of many cytokines and immunomodulatory factors that could exacerbate the progression of cutaneous forms of lupus. Of note, more than 1500 genes have been reported to be differentially expressed following UVR in skin.^25^ As the various modulators involved in the cutaneous response to UVR continue to be elucidated, some key agents and pathways that have been implicated in photosensitivity reactions are listed below.

LCs of the epidermis have been identified as playing a role in the development of photosensitive lesions in lupus erythematosus. LCs directly protect keratinocytes from UVR damage via an expression of an EGFR receptor that is activated by ADAM17, a metalloprotease. Without this interaction cells, human LCs experience increased photosensitivity. Patients with SLE show a reduced expression of EGFR receptors on LCs.^23^

The TNF‐like weak inducer of apoptosis (TWEAK) protein has also been implicated in photosensitivity.^26^ The sole receptor for TWEAK is induced by fibroblast growth factor inducible 14 (Fn14) in response to UVB radiation.^26^ Increased Fn14 receptor activation leads to increased apoptotic activity and macrophage recruitment. Following UVB radiation Fn14 knockout (Fn14KO) mice showed less hyperkeratosis, parakeratosis, disruption of the dermo–epidermal junction, and sunburn cells compared to wild‐type mice.^26^

Interferon genes, specifically type I interferon (IFN‐1), are found in elevated levels in the blood, kidneys, and skin of patients with SLE.^27^ IFN‐α‐producing plasmacytoid dendritic cells are present in CLE lesions, which may give an indication to the source of IFN‐1 in CLE.^28^ IFN‐α increases the autoantigen‐presenting abilities of monocytes/dendritic cells leading to the activation of autoreactive T‐cells.^29^ Interferon kappa (IFN‐κ) is a member of the type I interferon family and is primarily expressed by keratinocytes, and an overexpression of the IFNK gene can lead to autoimmune phenotypes in murine models.^28^ IFN‐κ mediates the upregulation of IFN‐1‐regulated gene expression activating dendrites in neighboring skin cells. IFN‐κ also regulates the apoptotic response to UVB and thus can be a target for the prevention of the progression of CLE sequelae.^28^, ^30^ Several trials of neutralizing IFN‐1 receptor monoclonal antibodies have been shown to cause significant improvements in clinical CLE, and a trial of monoclonal antibodies (sifalimumab) targeting IFN‐α showed slight improvement in patients with CLE.^31^, ^32^ However, monoclonal antibodies would likely not provide significant protection against all 13 IFN‐α subtypes.

The cytokine interleukin‐6 (IL‐6) is a well‐known mediator of systemic inflammation and is produced in excess by keratinocytes after exposure to UVB.^30^, ^33^ Patients with SLE produce significantly more IL‐6 compared to healthy controls after exposure to UVB and TLR‐2,‐3, or ‐4 agonists, an effect that is exacerbated by pretreatment with IFN‐α and IFN‐κ.^30^ Blocking IFN‐1 (which is mediated be IFN‐κ) has been shown to decrease IL‐6 production in lupus keratinocytes. Blockade of IL‐6 has also been shown to be therapeutic in murine models, but trials of IL‐6 antibody sirukumab failed to show clinical improvement for patients with SLE or CLE.^34^, ^35^

Extracellular vesicles (EVs) are 30–2000 nm^36^, ^37^, ^38^ subcellular structures containing phospholipid bilayer membranes that can be generated and released by essentially all cell types. Found in various biological fluids, EVs are highly heterogeneous and can be classified into three different subtypes based upon their size and biogenetic pathway. Exosomes (30–150 nm) are formed by the inward budding of endosomal membranes inside a cell. Microvesicles (MVs or microparticles) are 100–1500 nm in size and are pinched off from the external plasma membrane. Apoptotic bodies are 500–2000 nm in diameter and also are derived from the plasma membrane. The various types of EV exhibit considerable overlap in terms of sizes and compositions.

Several reports have documented increased EVs in the plasma of SLE patients.^39^, ^40^ In addition, a recent report identified small (200–700 nm) and large (700–3000 nm) EVs and showed that patients with active lupus nephritis had increased levels of large EVs containing mitochondria (mitoEVs) and IgG‐positive mitoEVs, suggesting the possibility that distinct EV subpopulations can exert different functions in the pathology of SLE.^39^ Regarding the function of these subcellular particles, it has been shown that these increased EVs in the plasma of SLE patients with active disease induced ROS production and degranulation in neutrophils,^41^ activated pDCs to release IFN‐α in a process involving the TLR7,^42^ or resulted in the senescence of mesenchymal stem cells associated with SLE.^43^

Though UVB has been shown to generate MVP in human keratinocytes, skin explants, as well as in skin and plasma of both mice and humans,^44^, ^45^, ^46^ the role of EV in photosensitivity is unclear. Yet studies at early (4–6 h) time points in cells, human skin explants, and mice do not appear to demonstrate increased exosomes produced by keratinocytes in response to UVB. Of note, the pathway of UVB‐induced MVP biogenesis has been characterized and includes the involvement of the lipid mediator platelet‐activating factor (PAF) and the enzyme acid sphingomyelinase.^37^ Hence, if MVPs are involved in the photosensitivity associated with SLE, then this would allow novel pharmacologic targets involving PAF and acid sphingomyelinase. Our group is currently examining whether preclinical models of photosensitivity involve MVP. As shown in Figure 1, unpublished preclinical studies using our published methodologies^44^ examined the ability of low UVB fluences to generate increased MVP in the skin and plasma reveal that fluences of UVB, which do not cause significant MVP generation in wild‐type mice, do so in a murine SLE mouse model NZM2328.^46^ Though this murine model of SLE has not been previously examined for photosensitivity, a recent report indicates that epidermal damage via tape stripping can promote nephritis.^46^, ^47^ The preliminary studies in Figure 1 suggest the possibility that subcellular particles could play an important role in the photosensitivity associated with SLE.

**FIGURE 1:**
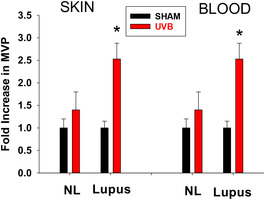
UVB treatment of NZM2328 lupus mice results in increased microvesicle particles (MVP) in skin and in plasma. Groups of 4–6 mice were treated with either 1000 J/m^2^ UVB or sham‐irradiated and 4 h later skin and plasma obtained or MVP measured.^44^ The data are Mean ± SE fold increase over untreated (sham). “*” denotes statistical significance (*p* < 0.05) compared to sham‐treated values by Student *t* test.

Treatments for cutaneous lesions related to LE involve advising patients to reduce sun exposure, especially between the hours of 10 AM and 4 PM, and utilize broad‐spectrum (UVA and UVB) SPF 50+ lotions. Sunscreens have been proven to prevent and reduce lesion formation in patients with SLE.^7^, ^13^, ^48^ Photo‐protective wear (long sleeves and pants, hats, and gloves) can reduce UVR access to the skin and should be promoted.^49^, ^50^ Avoiding the use of photosensitizing drugs in this population is also important. Supplemental vitamin D may be indicated in SLE patients, as vitamin D deficiency is common in those avoiding direct sunlight.^51^

Most treatments of cutaneous symptoms involve using pharmaceutical agents “off‐label.” Moreover, specific treatment protocols are not routinely established, which impacts adapted individualized care that is the optimal aim of treatment.^49^, ^50^ Topical corticosteroids are used for limited cutaneous disease, and systemic disease is generally treated with the antimalarial hydroxychloroquine. Cigarette smoking should be discouraged as it interferes with antimalarial efficacy.

Many stimuli exist that might trigger lesion formation in CLE and even induce systemic organ manifestation, with UVR, a common stressor often implicated.^52^, ^53^ Mediators of this damage may include MV's as their serum concentration increases following the photoprovocation of SLE. There is a need for continued study and characterization of the processes that lead to the rare systemic consequences of SLE after UVR exposure, as a rapid progression of the disease can occur.^53^ Elucidating inflammatory mediators as potential targets for drug therapy is an important consideration of our research. In particular, if subcellular particles such as MVP are involved, then pharmacologic strategies such as the use of acid sphingomyelinase inhibitors such as imipramine could be employed.^37^

Patient education concerning photosensitivity and lesions starts with uniform criteria for use by health‐care providers. A method of evaluating and characterizing patient sensitivity to UVR and the resulting lesion, rash, or systemic symptoms will help normalize the diagnostic process and will allow patients to better understand how symptomology affects their disease process. This clinical tool when paired with objective clinical markers will help with assessing disease progression. Ultimately, the management of CLE and SLE will be more feasible with the right tools to track early mediators of the disease. We propose the screening tool found in Figure 2 that consists of 10 questions to elicit evidence of photosensitivity.

**FIGURE 2:**
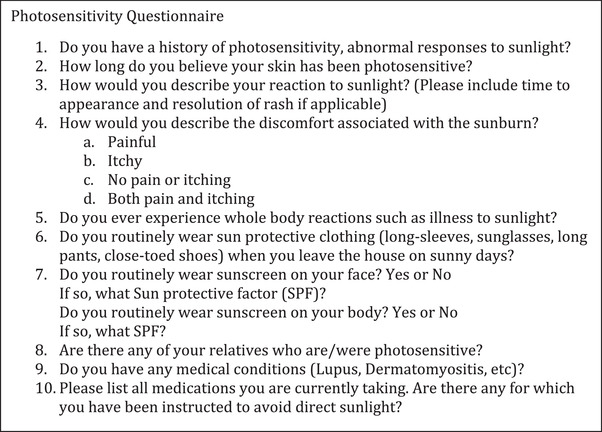
Photosensitivity screening questionnaire

Health‐care providers should take photos of eruptions and lesions when allowed and encourage patients to photograph these as well. Acquiring the information above from patients with photosensitive complaints can help physicians modify high‐risk behaviors and understand the patients’ understanding of their condition. The responses to this questionnaire can also function as a baseline for patients and are referred to as skin concerns change over time.

In patients with active lesions, biopsies and subsequent histopathologic staining provide insight to what inflammatory mediators characterize the disease process and are worth recording. Responses to the questionnaire in addition to a detailed patient history will help categorize photosensitive patient reactions to UVR as well as increase the documentation and histopathology of CLE lesions.

The definition of photosensitivity should be broadened due to the delayed effects of sun exposure specifically in patients with SLE. A unified method of assessing and reproducing photosensitive lesions will help structuralize clinical research and disease progression, ideally identifying promising treatment targets. Expanding our knowledge of immune modulators like MVP will aid in elucidating the pathways between UVR damage and organ system damage. Classifying the severity of photosensitive reactions can help physicians and patients treat and prevent lesion formation, as well as develop a standard of care for patients needing lifelong management.

## CONFLICTS OF INTEREST

The authors have no a conflict of interest.

## ETHICS STATEMENT

Animal studies were approved by the Wright State University Animal Committee.

## Data Availability

The data that support the findings of this study are available from the corresponding author upon reasonable request.

## References

[1] Fanouriakis A , Tziolos N , Bertsias G , Boumpas DT . Update Omicron the diagnosis and management of systemic lupus erythematosus. Ann Rheum Dis. 2021;80(1):14‐25. 33051219 10.1136/annrheumdis-2020-218272

[2] Kaul A , Gordon C , Crow MK , et al. Systemic lupus erythematosus. Nat Rev Dis Primers. 2016;2:16039. 27306639 10.1038/nrdp.2016.39

[3] Kim A , Chong BF . Photosensitivity in cutaneous lupus erythematosus. Photodermatol Photoimmunol Photomed. 2013;29(1):4‐11. 23281691 10.1111/phpp.12018 PMC3539182

[4] Aringer M , Costenbader K , Daikh D , et al. 2019 European League Against Rheumatism/American College of Rheumatology classification criteria for systemic lupus erythematosus. Arthritis Rheumatol. 2019;71(9):1400‐1412. 31385462 10.1002/art.40930 PMC6827566

[5] Dall'Era M , Cisternas MG , Snipes K , Herrinton LJ , Gordon C , Helmick CG . The incidence and prevalence of systemic lupus erythematosus in San Francisco County, California: the California lupus surveillance project. Arthritis Rheumatol. 2017;69(10):1996‐2005. 28891237 10.1002/art.40191

[6] Stojan G , Petri M . Epidemiology of systemic lupus erythematosus: an update. Curr Opin Rheumatol. 2018;30(2):144‐150. 29251660 10.1097/BOR.0000000000000480 PMC6026543

[7] Uva L , Miguel D , Pinheiro C , Freitas JP , Marques Gomes M , Filipe P . Cutaneous manifestations of systemic lupus erythematosus. Autoimmune Dis. 2012;2012:834291. 22888407 10.1155/2012/834291 PMC3410306

[8] Filotico R , Mastrandrea V . Cutaneous lupus erythematosus: clinico‐pathologic correlation. G Ital Dermatol Venereol. 2018;153(2):216‐229. 29368845 10.23736/S0392-0488.18.05929-1

[9] Okon LG , Werth VP . Cutaneous lupus erythematosus: diagnosis and treatment. Best Pract Res Clin Rheumatol. 2013;27(3):391‐404. 24238695 10.1016/j.berh.2013.07.008 PMC3927537

[10] Doglio M , Alexander T , Del Papa N , et al. New insights in systemic lupus erythematosus: from regulatory T cells to CAR‐T‐cell strategies. J Allergy Clin Immunol. 2022;S0091‐6749:01056‐01059. 10.1016/j.jaci.2022.08.003 36137815

[11] Klein B , Kunz M . Current concepts of photosensitivity in cutaneous lupus erythematosus. Front Med (Lausanne). 2022;9:939594. 36091671 10.3389/fmed.2022.939594 PMC9452788

[12] Kemp MG , Spandau DF , Simman R , Travers JB . Insulin‐like growth factor 1 receptor signaling is required for optimal ATR‐CHK1 kinase signaling in ultraviolet B (UVB)‐irradiated human keratinocytes. J Biol Chem. 2017;292(4):1231‐1239. 27979966 10.1074/jbc.M116.765883 PMC5270469

[13] Kuechle MK , Elkon KB . Shining light on lupus and UV. Arthritis Res Ther. 2007;9(1):101. 17284304 10.1186/ar2100 PMC1860055

[14] Li S , Jang GB , Quach C , Liang C . Darkening with UVRAG. Autophagy. 2019;15(2):366‐367. 30209981 10.1080/15548627.2018.1522911 PMC6333458

[15] Guzman E , Langowski JL , Owen‐Schaub L . Mad dogs, Englishmen and apoptosis: the role of cell death in UV‐induced skin cancer. Apoptosis. 2003;8(4):315‐325. 12815274 10.1023/a:1024112231953

[16] Kuhn A , Herrmann M , Kleber S , et al. Accumulation of apoptotic cells in the epidermis of patients with cutaneous lupus erythematosus after ultraviolet irradiation. Arthritis Rheum. 2006;54(3):939‐950. 16511837 10.1002/art.21658

[17] Wolf SJ , Estadt SN , Theros J , et al. Ultraviolet light induces increased T cell activation in lupus‐prone mice via type I IFN‐dependent inhibition of T regulatory cells. J Autoimmun. 2019;103:102291. 31248690 10.1016/j.jaut.2019.06.002 PMC6708464

[18] Kuhn A , Wenzel J , Weyd H . Photosensitivity, apoptosis, and cytokines in the pathogenesis of lupus erythematosus: a critical review. Clin Rev Allergy Immunol. 2014;47(2):148‐162. 24420508 10.1007/s12016-013-8403-x

[19] Chasset F , Frances C . Current concepts and future approaches in the treatment of cutaneous lupus erythematosus: a comprehensive review. Drugs. 2019;79(11):1199‐1215. 31228033 10.1007/s40265-019-01151-8

[20] Kuhn A , Sonntag M , Richter‐Hintz D , et al. Phototesting in lupus erythematosus: a 15‐year experience. J Am Acad Dermatol. 2001;45(1):86‐95. 11423840 10.1067/mjd.2001.114589

[21] Kuhn A , Sonntag M , Sunderkotter C , Lehmann P , Vestweber D , Ruzicka T . Upregulation of epidermal surface molecule expression in primary and ultraviolet‐induced lesions of lupus erythematosus tumidus. Br J Dermatol. 2002;146(5):801‐809. 12000376 10.1046/j.1365-2133.2002.04693.x

[22] Sanders CJ , Van Weelden H , Kazzaz GA , Sigurdsson V , Toonstra J , Bruijnzeel‐Koomen CA . Photosensitivity in patients with lupus erythematosus: a clinical and photobiological study of 100 patients using a prolonged phototest protocol. Br J Dermatol. 2003;149(1):131‐137. 12890206 10.1046/j.1365-2133.2003.05379.x

[23] Shipman WD , Chyou S , Ramanathan A , et al. A protective Langerhans cell‐keratinocyte axis that is dysfunctional in photosensitivity. Sci Transl Med. 2018;10(454):eaap9527. 30111646 10.1126/scitranslmed.aap9527 PMC6365282

[24] McGrath H Jr . Ultraviolet‐A1 irradiation therapy for systemic lupus erythematosus. Lupus. 2017;26(12):1239‐1251. 28480786 10.1177/0961203317707064 PMC5593127

[25] Kennedy Crispin M , Fuentes‐Duculan J , Gulati N , et al. Gene profiling of narrowband UVB‐induced skin injury defines cellular and molecular innate immune responses. J Invest Dermatol. 2013;133(3):692‐701. 23151847 10.1038/jid.2012.359 PMC3679916

[26] Doerner J , Chalmers SA , Friedman A , Putterman C . Fn14 deficiency protects lupus‐prone mice from histological lupus erythematosus‐like skin inflammation induced by ultraviolet light. Exp Dermatol. 2016;25(12):969‐976. 27305603 10.1111/exd.13108 PMC5127760

[27] Skopelja‐Gardner S , An J , Tai J , et al. The early local and systemic Type I interferon responses to ultraviolet B light exposure are cGAS dependent. Sci Rep. 2020;10(1):7908. 32404939 10.1038/s41598-020-64865-w PMC7220927

[28] Sarkar MK , Hile GA , Tsoi LC , et al. Photosensitivity and type I IFN responses in cutaneous lupus are driven by epidermal‐derived interferon kappa. Ann Rheum Dis. 2018;77(11):1653‐1664. 30021804 10.1136/annrheumdis-2018-213197 PMC6185784

[29] Lauwerys BR , Hachulla E , Spertini F , et al. Down‐regulation of interferon signature in systemic lupus erythematosus patients by active immunization with interferon alpha‐kinoid. Arthritis Rheum. 2013;65(2):447‐456. 23203821 10.1002/art.37785

[30] Stannard JN , Reed TJ , Myers E , et al. Lupus skin is primed for IL‐6 inflammatory responses through a keratinocyte‐mediated autocrine Type I interferon loop. J Invest Dermatol. 2017;137(1):115‐122. 27646883 10.1016/j.jid.2016.09.008 PMC5183476

[31] Greth W , Robbie GJ , Brohawn P , Hultquist M , Yao B . Targeting the interferon pathway with sifalimumab for the treatment of systemic lupus erythematosus. Immunotherapy. 2017;9(1):57‐70. 28000522 10.2217/imt-2016-0090

[32] Khamashta M , Merrill JT , Werth VP , et al. Sifalimumab, an anti‐interferon‐alpha monoclonal antibody, in moderate to severe systemic lupus erythematosus: a randomised, double‐blind, placebo‐controlled study. Ann Rheum Dis. 2016;75(11):1909‐1916. 27009916 10.1136/annrheumdis-2015-208562 PMC5099191

[33] Robinson ES , Werth VP . The role of cytokines in the pathogenesis of cutaneous lupus erythematosus. Cytokine. 2015;73(2):326‐334. 25767072 10.1016/j.cyto.2015.01.031

[34] Kuhn A , Ruland V , Bonsmann G . Cutaneous lupus erythematosus: update of therapeutic options part II. J Am Acad Dermatol. 2011;65(6):e195‐e213. 20800319 10.1016/j.jaad.2010.06.017

[35] Rovin BH , van Vollenhoven RF , Aranow C , et al. A multicenter, randomized, double‐blind, placebo‐controlled study to evaluate the efficacy and safety of treatment with sirukumab (CNTO 136) in patients with active lupus nephritis. Arthritis Rheumatol. 2016;68(9):2174‐2183. 27110697 10.1002/art.39722 PMC5129491

[36] Beck S , Hochreiter B , Schmid JA . Extracellular vesicles linking inflammation, cancer and thrombotic risks. Front Cell Dev Biol. 2022;10:859863. 35372327 10.3389/fcell.2022.859863 PMC8970602

[37] Frommeyer TC , Gilbert MM , Brittain GV , et al. UVB‐induced microvesicle particle release and its effects on the cutaneous microenvironment. Front Immunol. 2022;13:880850. 35603177 10.3389/fimmu.2022.880850 PMC9120817

[38] Ozkocak DC , Phan TK , Poon IKH . Translating extracellular vesicle packaging into therapeutic applications. Front Immunol. 2022;13:946422. 36045692 10.3389/fimmu.2022.946422 PMC9420853

[39] Mobarrez F , Fuzzi E , Gunnarsson I , et al. Microparticles in the blood of patients with SLE: size, content of mitochondria and role in circulating immune complexes. J Autoimmun. 2019;102:142‐149. 31103269 10.1016/j.jaut.2019.05.003

[40] Ullal AJ , Reich CF 3rd , Clowse M , et al. Microparticles as antigenic targets of antibodies to DNA and nucleosomes in systemic lupus erythematosus. J Autoimmun. 2011;36(3‐4):173‐180. 21376534 10.1016/j.jaut.2011.02.001

[41] Winberg LK , Jacobsen S , Nielsen CH . Microparticles from patients with systemic lupus erythematosus induce production of reactive oxygen species and degranulation of polymorphonuclear leukocytes. Arthritis Res Ther. 2017;19(1):230. 29041967 10.1186/s13075-017-1437-3 PMC5646131

[42] Ronnblom L , Alm GV , Eloranta ML . The type I interferon system in the development of lupus. Semin Immunol. 2011;23(2):113‐121. 21292501 10.1016/j.smim.2011.01.009

[43] Dong C , Zhou Q , Fu T , et al. Circulating exosomes derived‐miR‐146a from systemic lupus erythematosus patients regulates senescence of mesenchymal stem cells. Biomed Res Int. 2019;2019:6071308. 31428639 10.1155/2019/6071308 PMC6679864

[44] Liu L , Awoyemi AA , Fahy KE , et al. Keratinocyte‐derived microvesicle particles mediate ultraviolet B radiation‐induced systemic immunosuppression. J Clin Invest. 2021;131(10):e144963. 33830943 10.1172/JCI144963 PMC8121517

[45] Bihl JC , Rapp CM , Chen Y , Travers JB . UVB generates microvesicle particle release in part due to platelet‐activating factor signaling. Photochem Photobiol. 2016;92(3):503‐506. 26876152 10.1111/php.12577 PMC5426906

[46] Fahy K , Liu L , Rapp CM , et al. UVB‐generated microvesicle particles: a novel pathway by which a skin‐specific stimulus could exert systemic effects. Photochem Photobiol. 2017;93(4):937‐942. 28039861 10.1111/php.12703 PMC5494001

[47] Clark KL , Reed TJ , Wolf SJ , Lowe L , Hodgin JB , Kahlenberg JM . Epidermal injury promotes nephritis flare in lupus‐prone mice. J Autoimmun. 2015;65:38‐48. 26305061 10.1016/j.jaut.2015.08.005 PMC4679658

[48] Kuhn A , Gensch K , Haust M , et al. Photoprotective effects of a broad‐spectrum sunscreen in ultraviolet‐induced cutaneous lupus erythematosus: a randomized, vehicle‐controlled, double‐blind study. J Am Acad Dermatol. 2011;64(1):37‐48. 21167404 10.1016/j.jaad.2009.12.053

[49] Elhage KG , Zhao R , Nakamura M . Advancements in the treatment of cutaneous lupus erythematosus and dermatomyositis: a review of the literature. Clin Cosmet Investig Dermatol. 2022;15:1815‐1831. 10.2147/CCID.S382628 PMC9467686 36105749

[50] Kuhn A , Aberer E , Bata‐Csorgo Z , et al. S2k guideline for treatment of cutaneous lupus erythematosus – guided by the European Dermatology Forum (EDF) in cooperation with the European Academy of Dermatology and Venereology (EADV). J Eur Acad Dermatol Venereol. 2017;31(3):389‐404. 27859683 10.1111/jdv.14053

[51] Holick MF , Chen TC . Vitamin D deficiency: a worldwide problem with health consequences. Am J Clin Nutr. 2008;87(4):1080S‐1086S. 18400738 10.1093/ajcn/87.4.1080S

[52] Kreuter A , Lehmann P . Relevant new insights into the effects of photoprotection in cutaneous lupus erythematosus. Exp Dermatol. 2014;23(10):712‐713. 24910084 10.1111/exd.12466

[53] Schmidt E , Tony HP , Brocker EB , Kneitz C . Sun‐induced life‐threatening lupus nephritis. Ann N Y Acad Sci. 2007;1108:35‐40. 17893968 10.1196/annals.1422.004

